# Interleukin gene polymorphisms and breast cancer: a case control study and systematic literature review

**DOI:** 10.1186/1471-2407-6-188

**Published:** 2006-07-14

**Authors:** SP Balasubramanian, IAF Azmy, SE Higham, AG Wilson, SS Cross, A Cox, NJ Brown, MW Reed

**Affiliations:** 1 Academic Surgical Oncology Unit, University of Sheffield, Sheffield, UK; 2 Academic Rheumatology Unit, University of Sheffield, Sheffield, UK; 3 Academic Unit of Pathology, University of Sheffield, Sheffield, UK; 4 Institute of Cancer Studies, University of Sheffield, Sheffield, UK

## Abstract

**Background:**

Interleukins and cytokines play an important role in the pathogenesis of many solid cancers. Several single nucleotide polymorphisms (SNPs) identified in cytokine genes are thought to influence the expression or function of these proteins and many have been evaluated for their role in inflammatory disease and cancer predisposition. The aim of this study was to evaluate any role of specific SNPs in the interleukin genes IL1A, IL1B, IL1RN, IL4R, IL6 and IL10 in predisposition to breast cancer susceptibility and severity.

**Methods:**

Candidate single nucleotide polymorphisms (SNPs) in key cytokine genes were genotyped in breast cancer patients and in appropriate healthy volunteers who were similar in age, race and sex. Genotyping was performed using a high throughput allelic discrimination method. Data on clinico-pathological details and survival were collected. A systematic review of Medline English literature was done to retrieve previous studies of these polymorphisms in breast cancer.

**Results:**

None of the polymorphisms studied showed any overall predisposition to breast cancer susceptibility, severity or to time to death or occurrence of distant metastases. The results of the systematic review are summarised.

**Conclusion:**

Polymorphisms within key interleukin genes (IL1A, IL1B, IL1RN, IL4R, IL6 and IL10 do not appear to play a significant overall role in breast cancer susceptibility or severity.

## Background

The role of cytokines in cancer immunity and carcinogenesis in general has been well established [1]. Single nucleotide polymorphisms in specific candidate genes are thought to influence expression and/or activity of the encoding proteins thereby predisposing to solid cancers especially breast cancer [2]. Many cytokine polymorphisms have been studied for associations with susceptibility to gastric cancer [3-5], liver cancer [6,7], lung cancer[8], prostate cancer [9] and ovarian cancer [10] with mixed results.

The cytokines of the IL-1 family [11], IL-4 and its receptor [12,13], IL-6 [14,15] and IL-10 [16,17] are important candidate genes as they play an important role in breast cancer pathogenesis. IL1-alpha promotes growth of breast cancer cells and cachexia [18]. In breast cancer cells, IL1-beta increases the transcriptional activity of ER-alpha [19] which is a prognostic factor in breast cancer and the expression and stabilisation of IL-8 RNA [20] which is a potent angiogenic factor. IL-4 inhibits tumour growth by its anti-angiogenic effect [21] and inhibits growth and induces apoptosis of breast cancer cell lines in the presence of IL-4R [12]. Circulating IL6 levels have been found to be higher in breast cancer patients compared to healthy controls and among those with breast cancer, correlate with the stage of the disease [14]. IL10 is over expressed in breast tumours [16] and exogenous administration can mediate regression of tumour growth and breast cancer metastases in mice models [17].

The polymorphisms studied were selected in the light of previous reports of their effect on differential gene expression and/or disease susceptibility. The IL1A +4845 G>T polymorphism situated in exon 5 of the IL1A gene was described in 1993 [22] and results in an Ala to Ser amino acid substitution at residue 114 of the proIL1α molecule. Pro IL1α is cleaved between amino acids 112 and 113 and it has been suggested that this polymorphism may affect the proteolytic process [23]. The polymorphism is thought to influence C reactive protein levels in patients referred for coronary angiography [24] and influence the development of aggressive periodontitis in Chinese males [25]. Three polymorphisms commonly studied in the IL1B gene include -511 and -31 in the promoter region and the +3954 in exon 5, all representing a C>T single nucleotide change. The -511C>T and the +3954C>T SNPs are thought to influence C reactive protein levels in healthy individuals [26] and the +3954C>T polymorphism has been shown to influence IL1β production by monocytes *in vitro *[27]. The -511 polymorphism has been shown to be associated with vascular diseases such as stroke [28] and along with the +3954 polymorphisms has been extensively studied in gastric cancer [29-31]. The IL1RN +2018T>C polymorphism in exon 2 of the gene is in complete linkage disequilibrium with a penta-allelic 86 bp variable number of tandem repeat polymorphism in intron 2 of the gene which is strongly linked to increased production of IL1RA [32] and IL1β *in vitro *[33]. The penta-allelic polymorphism has been studied in several cancers including gastric cancer [29-31], lung cancer [34], ovarian cancer [35] and cervical cancer [36]. The +2018 SNP itself has been linked with Barrett's oesophagus and oesophageal cancer [37]. The IL4R 1902A>G polymorphism is an A to G transition at nucleotide 1902, causing a change in amino acid from glutamine to arginine at codon 576 in the interleukin-4 receptor alpha protein. This seems to alter the signalling function of the receptor, thereby predisposing carriers to disease [38]. Preliminary studies show some association of this polymorphism with Crohn's disease [39] and adult asthma [40]. The polymorphism has also been associated with an increased risk of renal cancer [41]. The IL6 -174G>C polymorphism in the 5' flanking region of the gene was initially reported in 1998 to influence IL6 expression and plasma levels (the -174C allele associated with lower expression and lower levels) [42]. Subsequent studies of this polymorphism show that the -174C allele decreases susceptibility to systemic juvenile idiopathic arthritis [43] and increases the risk of coronary artery disease presumably through inflammatory mechanisms [44,45]. It also has been shown to increase the risk of bladder cancer [46], colorectal cancer [47] and Kaposi's sarcoma in HIV infected men [48]. The IL10 -1082G>A polymorphism, situated in the promoter region of the gene, has been shown to influence IL10 protein production *in vitro *by concanavalin-A stimulated peripheral mononuclear cells [49]. The G allele is associated with an increased risk of Crohn's disease [50] and thought to increase predisposition to lung cancer [51]. The AA genotype has been shown to be associated with decreased survival in melanoma [52].

The aim of this study was to evaluate polymorphisms in specific cytokine genes [IL1A +4845G>T, IL1B -511C>T, IL1B +3954C>T, IL1RN +2018T>C, IL4R -1902A>G, IL6-174G>C and IL10-1082G>A] in a case control model to determine any associations with breast cancer susceptibility, severity and survival. A systematic review of the English language Medline literature through PubMed was performed to summarise all previous breast cancer related studies of the polymorphisms characterised in the current study.

## Methods

### Case-control study

The design and methodology of this case control study have previously been described [53,54]. Briefly, recruitment started in November 1998 and is ongoing. The cases include women diagnosed with breast cancer and being followed up at the Royal Hallamshire Hospital in Sheffield and Rotherham District General Hospital and controls were recruited from women attending the Sheffield Breast Screening Service. The study was restricted to white Caucasians, as there were insufficient individuals from other ethnic groups, for meaningful analysis. The South Sheffield Research Ethics Committee approved the study [Ref. no. SS98/137] and informed written consent was obtained from all subjects. Demographic, environmental risk factors and family history data were recorded for all breast cancer cases and mammography screening controls, using a standard questionnaire. Pathological data (including tumour grade, lymph node status and presence of vascular invasion) were obtained from medical records and validated by an experienced histopathologist (SSC). Data on disease recurrence and overall survival were obtained from the hospital records and the Trent Cancer Registry. The data was entered by trained personnel and stored in a Microsoft Access database and maintained by a dedicated database administrator. The data was validated for all the records (by SPB and database manager).

### Genotyping methods

Genomic DNA was extracted from frozen EDTA preserved peripheral venous blood from all individuals, as described previously [55]. The polymorphisms studied, along with the genes, location and unique ID is shown in Table 1. Genotyping of the polymorphisms was performed by the 5'nuclease PCR method, using the ABI/PE Biosystems Taqman™ system, essentially as described earlier [55]. Using specific primer and probe sequences (Table 1), PCR amplification was carried out separately for the different polymorphisms. The final concentrations of the different constituents of the PCR mixture and the cycling temperatures for the various SNPs studied are shown in Tables 2 and 3. Levels of FAM and TET fluorescence were determined and allelic discrimination was carried out using the ABI 7200 Sequence Detector. Quality control for the genotyping results was achieved by using only 72 of the 96 wells in each of the plates for the individual DNA samples subjected to PCR. Six to eight wells were allotted to 'no sample' controls, 'common homozygous' controls and 'rare homozygous' controls each, in addition to retesting of samples with indeterminate results. The common and rare homozygous controls included samples tested before and shown to be 'common homozygous' and 'rare homozygous' respectively.

### Methodology for systematic review

A Medline search was conducted on 26^th ^September 2005 with the following search strategy: (("interleukins"[TIAB] NOT Medline [SB]) OR "interleukins"[MeSH Terms] OR interleukin[Text Word]) OR (("cytokines"[TIAB] NOT Medline[SB]) OR "cytokines"[MeSH Terms] OR cytokine[Text Word]) AND ("genetic polymorphism"[Text Word] OR "polymorphism, genetic"[MeSH Terms] OR polymorphism[Text Word]) OR SNP[All Fields] AND (("neoplasms"[TIAB] NOT Medline[SB]) OR "neoplasms"[MeSH Terms] OR cancer[Text Word]) AND English[Lang]. Only articles on the polymorphisms evaluated in this study were included for the purposes of the review and their results are summarised in the discussion.

### Data processing and analysis

All data were entered initially into a Microsoft Access database and exported to SPSS (version 12.0.1 for Windows) for statistical analyses. Chi-square test for trend was performed to compare the genotype frequencies (1:1, 1:2 and 2:2 representing the common homozygous, heterozygous and the rare heterozygous respectively) between cases and controls and also for comparison of the genotype frequencies among the various subgroups of breast cancer. Kaplan Meier curves and the log rank test was used for the survival analyses. All tests were two sided. Haplotype analysis was then performed on the genotype data of the four polymorphisms (IL1A +4845G>T, IL1B +3954C>T, IL1B -511C>T and IL1RN +2018T>C) in chromosomal region 2q13 using Haploview [56].

## Results

The demographic characteristics and comparability of case and control cohorts have been reported previously [53,54]. Briefly, the case and control groups were all Caucasian and female. There were no significant differences in the percentage of postmenopausal women, age at menarche and age at menopause between the cancer and control groups. The women in the control groups were however younger [median (IQR) of 57 (53–61) in the control group vs. 63 (54–70) in the cancer group; p < 0.001; Mann-Whitney U test], were younger when first pregnant [median (IQR) of 23 (20–26) in the control group vs. 24 (21–27) in the cancer group; p < 0.001; Mann-Whitney U test], had more children [median (IQR) of 2 (2–3) in the control group vs. 2 (1–3) in the cancer group; p < 0.001; Mann-Whitney U test], were less likely to have a family history of breast cancer [22.2% in controls vs. 27.4% in cancers; p = 0.007; Chi-square test] and were more likely not to have smoked [63.1% in controls vs. 53.4% in cancers; p < 0.001; Chi-square test].

Table 4 shows the total numbers, the observed frequencies and the expected genotype frequencies (expected genotype frequencies were calculated from the respective allele frequencies) in the control population and the testing for the Hardy Weinberg Equilibrium. The observed frequencies of the genotypes for all polymorphisms are not significantly different from the expected frequencies except for the IL1A +4845 and the IL4R +1902 polymorphisms.

**Figure 1:**
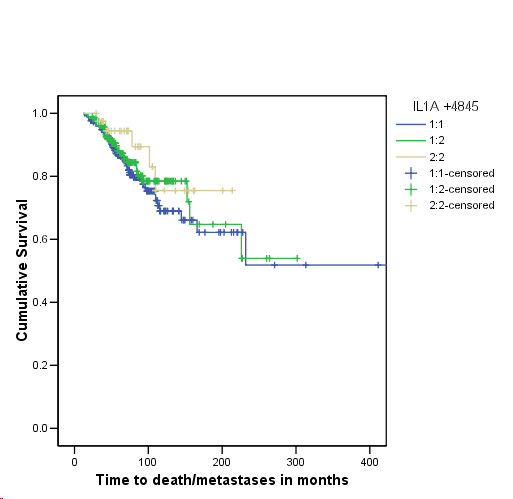
shows the time to death or metastases in invasive breast cancer for the genotypes of the IL1A +4845 polymorphism. Log Rank test statistic = 1.52; df = 2; p = 0.47 (n = 482).

**Figure 2:**
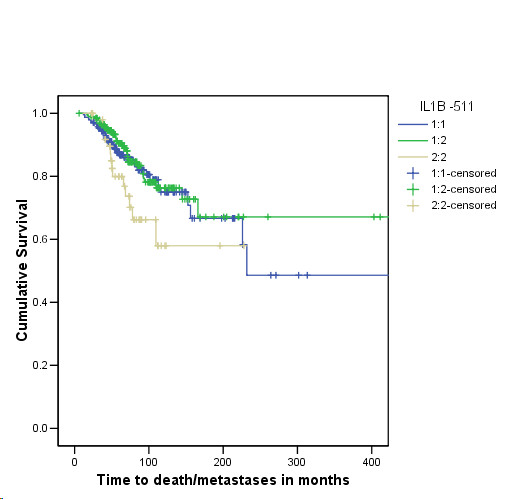
shows the time to death or metastases in invasive breast cancer for the genotypes of the IL1B -511 polymorphism. Log Rank test statistic = 5.07; df = 2; p = 0.08 (n = 484).

**Figure 3:**
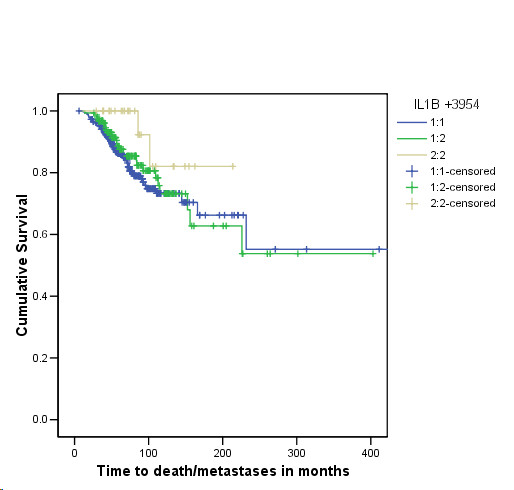
shows the time to death or metastases in invasive breast cancer for the genotypes of the IL1B +3954 polymorphism. Log Rank test statistic = 2.71; df = 2; p = 0.26 (n = 479).

**Figure 4:**
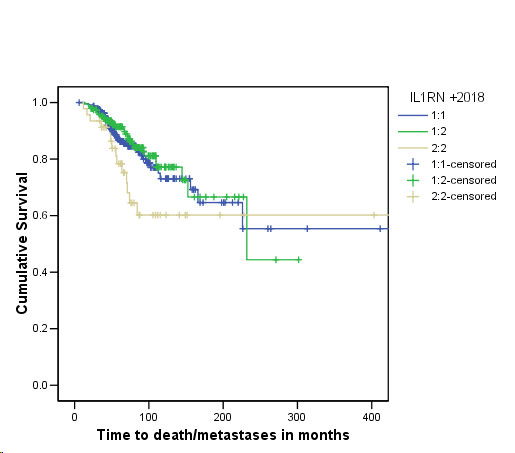
shows the time to death or metastases in invasive breast cancer for the genotypes of the IL1RN +2018 polymorphism. Log Rank test statistic = 4.32; df = 2; p = 0.12 (n = 481).

**Figure 5:**
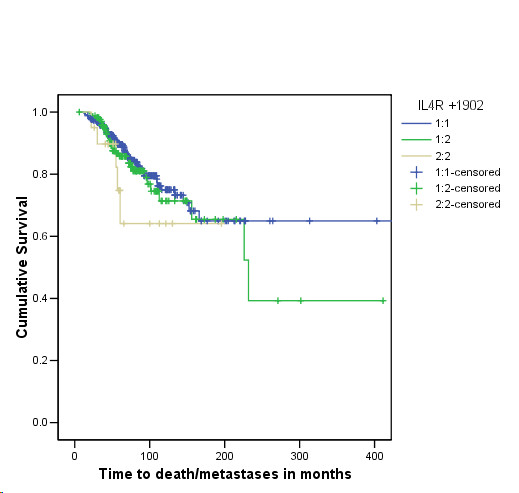
shows the time to death or metastases in invasive breast cancer for the genotypes of the IL4R +1902 polymorphism. Log Rank test statistic = 2.07; df = 2; p = 0.35 (n = 528).

**Figure 6:**
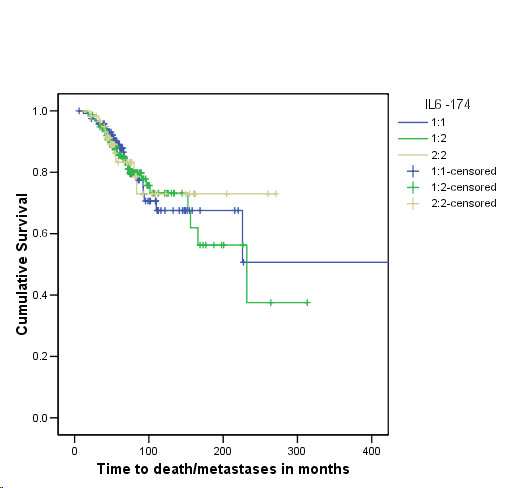
shows the time to death or metastases in invasive breast cancer for the genotypes of the IL6 -174 polymorphism. Log Rank test statistic = 0.16; df = 2; p = 0.92 (n = 333).

**Figure 7:**
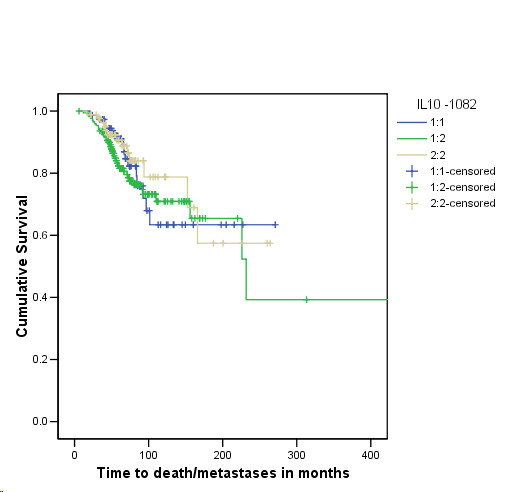
shows the time to death or metastases in invasive breast cancer for the genotypes of the IL10 -1082 polymorphism. Log Rank test statistic = 1.34; df = 2; p = 0.51 (n = 332).

**Figure 8:**
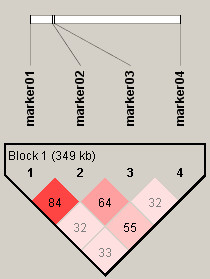
shows the linkage disequilibrium plot (obtained using Haploview) showing pairwise D' values (in percentage) between the four polymorphisms on chromosome 2q13. The markers 1, 2, 3 and 4 are IL1A +4845G>T, IL1B +3954C>T, IL1B -511 C>T and IL1RN +2018T>C respectively.

The comparison of genotype frequencies between the control and cancer groups for each of the polymorphisms (along with the actual numbers studied) are shown in Table 5. In addition to overall comparisons, the genotype frequencies were compared in subgroups classified according to family history and age at diagnosis. Table 6 shows the genotype frequencies for the seven polymorphisms within subgroups of invasive breast cancer (defined by tumour grade, nodal status and vascular invasion). Figures 1, 2, 3, 4, 5, 6, 7, 8 show survival curves demonstrating that none of the polymorphisms had any impact on time to death or development of metastases in those with invasive breast cancer.

Further analyses of the four polymorphisms in the Interleukin-1 gene cluster (IL1A +4845G>T, IL1B +3954C>T, IL1B -511C>T and IL1RN +2018T>C) were done using Haploview. These four polymorphisms are situated a region of size 360 kb. The LD (linkage disequilibrium) values for the four pairs of SNPs (Figure 8) and the probable haplotypes with their frequencies (Table 7) have been calculated. None of the estimated haplotypes was associated with breast cancer in this cohort.

The literature search demonstrated two previous studies on the IL1B -511C>T polymorphism [57,58], one on the IL1B +3954C>T polymorphism [58], six on the IL6 -174G>C polymorphism Smith, 2004 #877} [58-62] and four on the IL10 -1082G>A polymorphism [57,59,63,64]. The results of the previously published studies are discussed in the context of the results from the current study in the next section.

## Discussion

Cytokines play varied roles in cancer pathogenesis with increasing evidence suggesting their involvement in tumour initiation, growth and metastasis [1]. Cytokine gene polymorphisms have been studied for associations with many inflammatory and neoplastic diseases. Numerous reports have evaluated the association of individual candidate SNPs in cytokine genes in breast cancer, some of which are included in this study.

### IL1A polymorphisms and breast cancer

IL1A is thought to contribute to breast cancer expression by up-regulating pro-metastatic genes in breast cancer cells and stromal cells [65]. IL1A levels in breast tissue homogenates correlates inversely with ER levels [66], which is an established prognostic marker in breast cancer. The IL1A gene is mapped to chromosome 2q13 and includes several polymorphisms, of which one in the 5'UTR regulatory region (-889C>T) and one in exon 5 of the gene (+4845G>T) have been commonly studied. The IL1A -889 polymorphism has been studied in two different cohorts and not shown to be associated with breast cancer [58,67]. However, to date, there are no published studies on the role of the IL1A +4845 polymorphism in breast cancer. The current study has shown that there is a trend for the rare allele to confer a protective effect against cancer (p = 0.05) and for the common allele to be significantly associated with lymph node positive cancers (p = 0.03). This effect is more apparent when the rare allele carriage rates (carriers of rare alleles) are assessed instead of genotype frequencies (p = 0.005 and p = 0.007 respectively). The positive finding however has not been subject to corrections for multiple testing in view of the exploratory nature of these studies. In addition, given that the genotype frequencies of this polymorphism were not in Hardy Weinberg equilibrium, this may be an artefactual association which would need confirmation in other populations. There was no association of this polymorphism with tumour grade, vessel invasion or survival.

### IL1B polymorphisms and breast cancer

IL1β levels are high in breast cancer tissue and correlate with invasiveness and an aggressive phenotype [68]. They seem to regulate cancer cell proliferation through oestrogen production by steroid-catalyzing enzymes in the tissue [69]. The IL1B gene is mapped to 2q13 [70] and the commonly described genetic variants include the -511C>T and the -31C>T in the 5'UTR and the +3954C>T polymorphism in exon 5 of the gene. Our data for the -511 and the +3954 SNPs show that overall; neither of these SNPs is associated with breast cancer susceptibility, severity or survival. As table 4c shows, in women with a positive family history of breast cancer, the IL1B +3954T allele was associated with a reduced risk of breast cancer. The significance of this association on exploratory subgroup analysis is however limited. Tables 8 and 9 show data from two other studies confirming our findings that these polymorphisms do play a significant role in breast cancer susceptibility or severity.

### IL1RN polymorphisms and breast cancer

It has been shown that IL1RA levels are increased in breast cancer tissue and that IL1RA levels correlate with ER levels [66]. At least 18 sequence variants exist around the IL1RN gene [71] located in chromosome 2q13 [70]. Of these, the penta-allelic variant in intron 2 and the +2018T>C have been commonly studied. There are no prior reports of the IL1RN +2018 polymorphism in breast cancer. The intronic polymorphism described however has however been studied in breast cancer without any significant association with susceptibility or prognosis [58]. Our data shows no association of the +2018T>C polymorphism with breast cancer risk, severity or survival from the disease.

In addition to the analysis of the individual polymorphisms in the IL1A, IL1B and IL1RN genes, comparison of the probable haplotype frequencies in the breast cancer and control cohorts did not show any significant differences between the two groups.

### IL4R polymorphisms and breast cancer

IL4 receptor is significantly expressed in breast cancer [72] and it has been shown that IL4R is required for actions of IL4 on breast cancer cells [12] including the inhibition of growth and induction of apoptosis. The IL4R gene has been localised to 16p12. Several coding and regulatory region polymorphisms exist in the IL4R gene and are thought to influence signal transduction on the IL4 signalling pathway [73]. Our data on the IL4R polymorphism +1902A>G has shown no overall association with breast cancer susceptibility, severity or survival. In the subgroup of young cancer patients (those </=50 years at diagnosis), we found that the G allele was significantly associated with breast cancer. There are no other studies of this polymorphism in breast cancer.

### IL6 polymorphisms and breast cancer

The circulating level of interleukin 6 is thought to be elevated in the development and progression of many tumours including breast cancer and its up-regulation is associated with invasiveness and increased metastatic potential of ER negative tumours [74]. The IL6 gene has been localised to chromosome 7p21. Although several polymorphisms exist in the promoter region of IL-6 and are thought to have a complex interactive effect on IL6 expression [75], the polymorphism at -174 has been most extensively studied and shown to have significant ethnic variation [74]. Table 10 shows the various studies of this polymorphism in breast cancer to date. Only one study demonstrated an association with breast cancer susceptibility [58], which showed a significant Odds ratio of 1.5 and 2.0 for the heterozygotes (GC) and the rare homozygotes (CC) when compared to the common homozygotes (GG). The study however included a non-healthy control population (women attending outpatient departments for various reasons) and a lack of correction for multiple testing. Our data shows that the IL6 -174G>C polymorphism was not associated with either breast cancer risk or severity and prognosis as assessed by tumour grade, lymph nodal status, vascular invasion or survival.

### IL10 polymorphisms and breast cancer

IL10 has been shown to have anti-metastatic and anti-tumour effects in murine breast cancer models [17]. Mononuclear cells from breast cancer patients exhibit increased IL10 production [76] and IL10 serum levels correlate with stage of the disease [77]. Several single nucleotide polymorphisms exist in the promoter region of the IL10 gene (localised to chromosome 1q31-q32) including -1082, -819 and -592 [78]. Table 11 shows the studies of the IL10 -1082G>A polymorphism in breast cancer. Of the three studies reported, only one suggests a role for the G allele in reducing breast cancer risk [63]. Our data, which includes larger numbers of individuals, however shows no association with breast cancer susceptibility, severity or survival for this polymorphism. A study on the related polymorphism (-592C>A) in the promoter region was associated with a reduced breast cancer risk, although in breast cancer patients, there was no association with severity of the disease [79].

## Limitations

Although our study had more than twice the number of subjects than the similar studies on cytokine polymorphisms in breast cancer, it could still be argued that associations of a minor degree (Odds Ratio < 1.5) of the genetic markers studied or other related markers may have been missed. For example, to detect a rare marker (of frequency 10%) associated with a 1.3 times increased risk of breast cancer (Odds Ratio = 1.3) with a power of 80% and type I error of 0.5%, we would need a sample size of 2400 patients and controls. The second limitation is that a small proportion of our control population would develop breast cancer in their lifetime. However, it is generally considered difficult to obtain an ideal control cohort for genetic epidemiologic studies in solid cancers mainly due to the delayed onset of the disease. The prognostic markers used for assessing breast cancer severity in this study were limited to grade, lymph nodal status and vascular invasion due to limited information available on other indices such as hormone receptor status and tumour size.

## Conclusion

The results from our study do not support the hypothesis that the cytokine polymorphisms studied [IL1A +4845G>T, IL1B -511C>T, IL1B +3954C>T, IL1RN +2018T>C, IL4R -1902A>G, IL6-174G>C and IL10-1082G>A] are associated with breast cancer susceptibility and severity. Minor influences and associations with subgroups of phenotypes may exist, but are unlikely to be of any major clinical significance.

## Abbreviations

IL: interleukin; ER: oestrogen receptor; SNP: single nucleotide polymorphism; UTR: untranslated region; PCR: polymerase chain reaction; DNA: deoxyribonucleic acid.

## Competing interests

The author(s) declare that they have no competing interests.

## Authors' contributions

SPB, IAFA and SEH carried out patient recruitment, the molecular genetic studies and drafted the manuscript. SSC reviewed the pathology and drafted the manuscript. SPB and AC participated in the design of the study and performed the statistical analysis. AGW, NJB and MWR conceived of the study, and participated in its design and coordination and helped to draft the manuscript. All authors read and approved the final manuscript.

## Pre-publication history

The pre-publication history for this paper can be accessed here:



## Figures and Tables

**Table 1:** Candidate single nucleotide polymorphisms (SNPs) and their respective probes and primers

**Gene**	**Location**	**SNP ID**	**Forward primer**	**Reverse primer**	**FAM probe**	**TET probe**
IL-1A	+4845 G>T	rs17561	TGCACTTGTGATCATGGTTTTAGA	TCCTCATAAAGTTGTATTTCACATTGC	CAAGCCTAGGTCA**T**CACCTTTTAGCTTCC	AAGCCTAGGTCA**G**CACCTTTTAGCTTCC
IL-1B	-511 C>T	rs16944	TTGAGGGTGTGGGTCTCTACCT	AGGAGCCTGAACCCTGCATAC	TTCTCTGCCTC**G**GGAGCTCTCTGT	TTCTCTGCCTC**A**GGAGCTCTCTGTCA
IL-1B	+3954 C>T	rs1143634	GCCTGCCCTTCTGATTTTATACC	CATCGTGCACATAAGCCTCGTTA	TTCAGAACCTATCTTCTT**T**GACACATGGGA	CAGAACCTATCTTCTT**C**GACACATGGGA
IL-1RN	+2018 T>C	rs419598	GGGATGTTAACCAGAAGACCTTCTATCT	CAACCACTCACCTTCTAAATTGACATT	AACAACCAACTAGTTGC**T**GGATACTTGCAA	ACAACCAACTAGTTGC**C**GGATACTTGC
IL-4R	+1902 A>G	rs1801275	AGGCTTGAGAAGGCCTTGTAA	CCGAAATGTCCTCCAGCAT	CATGTACAAACTCC**T**GATAGCCACTGGTG	CATGTACAAACTCC**C**GATAGCCACTGG
IL-6	-174 G>C	rs1800795	GCTGATTGGAAACCTTATTAAGATTGT	AATGACGACCTAAGCTGCACTTT	ACGTCCTTTAGCAT**C**GCAAGACACAAC	ACGTCCTTTAGCAT**G**GCAAGACACAAC
IL-10	-1082 G>A	rs1800896	GATAGGAGGTCCCTTACTTTCCTCTTA	CACACACAAATCCAAGACAACACTAC	CTACTTCCCC**C**TCCCAAAGAAGCCT	CCTACTTCCCC**T**TCCCAAAGAAGCC

Note: All sequences are from 5' end to 3' end.

**Table 2:** Final concentration of the different constituents of the PCR mixture

**PCR constituents**	**Final concentrations for the various SNPs**						
	
	**IL1A +4845**	**IL1B -511**	**IL1B +3954**	**IL1RN +2018**	**IL4R +1902**	**IL6 -174**	**IL10 -1082**
Taqman mastermix (2X)	1X	1X	1X	1X	1X	1X	1X
Forward primer (10 μM)	500 nM	100 nM	300 nM	250 nM	50 nM	50 nM	50 nM
Reverse primer (10 μM)	500 nM	100 nM	300 nM	250 nM	500 nM	50 nM	300 nM
FAM probe (5 μM)	50 nM	50 nM	50 nM	30 nM	30 nM	30 nM	50 nM
TET probe (5 μM)	100 nM	100 nM	75 nM	150 nM	120 nM	60 nM	150 nM
Template (20 ng/μl)	0.8 ng/μl	0.8 ng/μl	0.8 ng/μl	0.8 ng/μl	0.8 ng/μl	0.8 ng/μl	0.8 ng/μl

Taqman mastermix: Universal PCR mastermix (PE Biosystems) containing MgCl_2_, dNTPs, Taq polymerase, optimised buffer components and Rox reference dye; FAM probe: 6-carboxy-fluorescein-labelled probe; TET: 6-carboxy-4,7,2',7'-tetrechlorofluorescein-labelled probe.

**Table 3:** Cycling conditions for the PCRs for the different polymorphisms

**Steps**	**Time**	**Temperature for the various SNPs**						
		
		**IL1A +4845**	**IL1B -511**	**IL1B +3954**	**IL1RN +2018**	**IL4R +1902**	**IL6 -174**	**IL10 -1082**
1	2 minutes	50°C	50°C	50°C	50°C	50°C	50°C	50°C
2	10 minutes	95°C	95°C	95°C	95°C	95°C	95°C	95°C
3	15 seconds	95°C	95°C	95°C	95°C	95°C	95°C	95°C
4	1 minutes	**59°C**	**59°C**	**61°C**	**64°C**	**61°C**	**62°C**	**62°C**
5	40 times	Go to step 3	Go to step 3	Go to step 3	Go to step 3	Go to step 3	Go to step 3	Go to step 3
6	Hold	15°C	15°C	15°C	15°C	15°C	15°C	15°C

**Table 4:** Observed and Expected genotype frequencies and the HardyWeinberg Equilibrium in the control population

**SNP**	**Controls (n)**	**Observed Genotype Frequency**			**Allele Frequency (in %)**		**Expected Genotype Frequency**		**Chi-square Goodness of fit test statistic (p value)**	
		
		**1:1**	**1:2**	**2:2**	**1**	**2**	**1:1**	**1:2**	**2:2**	**2:2**
**IL1A +4845**	498	215	245	38	67.8	32.2	229	217	52	χ^2 ^= 7.49; p = 0.01
**IL1B -511**	489	232	206	51	66.5	31.5	230	211	48	χ^2 ^= 0.20; p = 0.66
**IL1B +3954**	420	231	167	22	74.9	25.1	235	158	27	χ^2 ^= 1.13; p = 0.29
**IL1RN +2018**	490	247	202	41	71	29	247	202	41	χ^2 ^= 0; p = 0.95
**IL4R +1902**	767	451	288	28	77.6	22.4	461	267	39	χ^2 ^= 4.45; p = 0.03
**IL6 -174**	490	168	235	87	58.3	41.7	167	238	85	χ^2 ^= 0.06; p = 0.81
**IL10 -1082**	498	117	260	121	49.6	50.4	123	249	126	χ^2 ^= 0.85; p = 0.36

**Table 5:** Genotype frequencies of the seven polymorphisms in subgroups of breast cancer and control populations

**Subsets**	**Case/control**	**Genotype Frequencies (%)**			**Chi square test for trend (p value)**
			
		1:1	1:2	2:2	
**IL1A +4845 G>T**					

**Overall**	Cancers (n = 697)	360 (51.6%)	275 (39.5%)	62 (8.9%)	X^2 ^= 3.71; p = 0.05
	Controls (n = 498)	215 (43.2%)	245 (49.2%)	38 (7.6%)	
**Positive Family History**	Cancers (n = 192)	106 (55.2%)	71 (37%)	15 (7.8%)	X^2 ^= 2.74; p = 0.10
	Controls (n = 104)	44 (42.3%)	52 (50%)	8 (7.7%)	
**Negative Family History**	Cancers (n = 505)	254 (50.3%)	204 (40.4%)	47 (9.3%)	X^2 ^= 1.47; p = 0.23
	Controls (n = 394)	171 (43.4%)	193 (49%)	30 (7.6%)	
**Young cancers vs. controls**	Cancers (n = 113)	55 (48.7%)	43 (38%)	15 (13.3%)	X^2 ^= 0; p = 0.98
	Controls (n = 498)	215 (43.2%)	245 (49.2%)	38 (7.6%)	

**IL1B -511 C>T**					

**Overall**	Cancers (n = 703)	339 (48.2%)	294 (41.8%)	70 (10%)	X^2 ^= 0.10; p = 0.75
	Controls (n = 489)	232 (47.4%)	206 (42.1%)	51 (10.4%)	
**Positive Family History**	Cancers (n = 195)	96 (49.2%)	85 (43.6%)	14 (7.2%)	X^2 ^= 0.45; p = 0.51
	Controls (n = 103)	48 (46.6%)	45 (43.7%)	10 (9.7%)	
**Negative Family History**	Cancers (n = 508)	243 (47.8%)	209 (41.1%)	56 (11%)	X^2 ^= 0.003; p = 0.96
	Controls (n = 386)	184 (47.7%)	161 (41.7%)	41 (10.6%)	
**Young cancers vs. controls**	Cancers (n = 115)	55 (47.8%)	49 (42.6%)	11 (9.6%)	X^2 ^= 0.033; p = 0.86
	Controls (n = 489)	232 (47.4%)	206 (42.1%)	51 (10.4%)	

**IL1B +3954 C>T**					

**Overall**	Cancers (n = 691)	410 (59.3%)	242 (35%)	39 (5.6%)	X^2 ^= 1.12; p = 0.29
	Controls (n = 420)	231 (55%)	167 (39.8%)	22 (5.2%)	
**Positive Family History**	Cancers (n = 193)	129 (66.8%)	55 (28.5%)	9 (4.7%)	X^2 ^= 8.75; p = 0.003*
	Controls (n = 91)	43 (47.3%)	41 (45.1%)	7 (7.7%)	
**Negative Family History**	Cancers (n = 498)	281 (56.4%)	187 (37.6%)	30 (6%)	X^2 ^= 0.26; p = 0.61
	Controls (n = 329)	188 (57.1%)	126 (38.3%)	15 (4.6%)	
**Young cancers vs. controls**	Cancers (n = 112)	64 (57.1%)	41 (36.6%)	7 (6.3%)	X^2 ^= 0.03; p = 0.86
	Controls (n = 420)	231 (55%)	167 (39.8%)	22 (5.2%)	

**IL1RN +2018 T>C**					

**Overall**	Cancers (n = 697)	349 (50.1%)	286 (41%)	62 (8.9%)	X^2 ^= 0.05; p = 0.82
	Controls (n = 490)	247 (50.4%)	202 (41.2%)	41 (8.4%)	
**Positive Family History**	Cancers (n = 193)	94 (48.7%)	84 (43.5%)	15 (7.8%)	X^2 ^= 0.074; p = 0.79
	Controls (n = 103)	48 (46.6%)	47 (45.6%)	8 (7.8%)	
**Negative Family History**	Cancers (n = 504)	255 (50.6%)	202 (40.1%)	47 (9.3%)	X^2 ^= 0.14; p = 0.71
	Controls (n = 387)	199 (51.4%)	155 (40.1%)	33 (8.5%)	
**Young cancers vs. controls**	Cancers (n = 113)	61 (54%)	44 (38.9%)	8 (7.1%)	X^2 ^= 0.53; p = 0.47
	Controls (n = 490)	247 (50.4%)	202 (41.2%)	41 (8.4%)	

**IL4R +1902 A>G**					

**Overall**	Cancers (n = 775)	493 (63.6%)	249 (32.1%)	33 (4.3%)	X^2 ^= 2.1; p = 0.15
	Controls (n = 767)	451 (58.8%)	288 (37.5%)	28 (3.7%)	
**Positive Family History**	Cancers (n = 212)	133 (62.7%)	70 (33%)	9 (4.2%)	X^2 ^= 0.19; p = 0.66
	Controls (n = 168)	98 (58.3%)	66 (39.3%)	4 (2.4%)	
**Negative Family History**	Cancers (n = 563)	360 (63.9%)	179 (31.8%)	24 (4.3%)	X^2 ^= 2.00; p = 0.16
	Controls (n = 599)	353 (58.9%)	222 (37.1%)	24 (4.0%)	
**Young cancers vs. controls**	Cancers (n = 122)	85 (69.7%)	36 (29.5%)	1 (0.8%)	X^2 ^= 6.36; p = 0.012*
	Controls (n = 767)	451 (58.8%)	288 (37.5%)	28 (3.7%)	

**IL6 -174 G>C**					

**Overall**	Cancers (n = 497)	170 (34.2%)	244 (49.1%)	83 (16.7%)	X^2 ^= 0.05; p = 0.83
	Controls (n = 490)	168 (34.3%)	235 (48%)	87 (17.8%)	
**Positive Family History**	Cancers (n = 127)	47 (37%)	55 (43.3%)	25(19.7%)	X^2 ^= 0.20; p = 0.66
	Controls (n = 102)	38 (37.3%)	48 (47.1%)	16(15.7%)	
**Negative Family History**	Cancers (n = 370)	123 (33.2%)	189 (51.1%)	58 (15.7%)	X^2 ^= 1.22; p = 0.64
	Controls (n = 388)	130 (33.5%)	187 (48.2%)	71 (18.3%)	
**Young cancers vs. controls**	Cancers (n = 85)	36 (42.4%)	31 (36.5%)	18 (21.2%)	X^2 ^= 0.31; p = 0.58
	Controls (n = 490)	168 (34.3%)	235 (48%)	87 (17.8%)	

**IL10 -1082 G>A**					

**Overall**	Cancers (n = 497)	121 (24.3%)	253 (50.9%)	123 (24.7%)	X^2 ^= 0.01; p = 0.93
	Controls (n = 498)	117 (23.5%)	260 (52.2%)	121 (24.3%)	
**Positive Family History**	Cancers (n = 126)	31 (24.6%)	69 (54.8%)	26 (20.6%)	X^2 ^= 0.39; p = 0.54
	Controls (n = 104)	31 (29.8%)	52 (50%)	21 (20.2%)	
**Negative Family History**	Cancers (n = 371)	90 (24.3%)	184 (49.6%)	97 (26.1%)	X^2 ^= 0.11; p = 0.74
	Controls (n = 394)	86 (21.8%)	208 (52.8%)	100 (25.4%)	
**Young cancers vs. controls**	Cancers (n = 84)	17 (20.2%)	44 (52.4%)	23 (27.4%)	X^2 ^= 0.60; p = 0.44
	Controls (n = 498)	117 (23.5%)	260 (52.2%)	121 (24.3%)	

**Note**: Family history: either first or second degree relative with breast cancer. Young cancer patients: </=50 years of age

**Table 6:** Genotype frequencies of the seven polymorphisms in subgroups of invasive breast cancer.

**Tumour Severity**	**Subgroups**	**Genotype frequencies (%)**			**Chi square test for trend (P value)**
			
		1:1	1:2	2:2	
**IL1A +4845 G>T**					

**Tumour Grade**	Grade 1 (n = 122)	62 (50.8%)	50 (41%)	10 (8.2%)	X^2 ^= 0.037; p = 0.85
	Grade 2 (n = 283)	151 (53.4%)	109 (38.5%)	23 (8.1%)	
	Grade 3 (n = 216)	115 (53.2%)	82 (38%)	19 (8.8%)	
**Nodal Involvement**	Absent (n = 430)	204 (47.4%)	118 (43.7%)	38 (8.8%)	X^2 ^= 4.75; p = 0.03*
	Present (n = 117)	117 (59.4%)	63 (32%)	17 (8.6%)	
**Vascular Invasion**	Absent (n = 467)	243 (52%)	185 (39.6%)	39 (8.4%)	X^2 ^= 0.058; p = 0.81
	Present (n = 117)	64 (54.7%)	42 (35.9%)	11 (9.4%)	

**IL1B -511 C>T**					

**Tumour Grade**	Grade 1 (n = 126)	51 (40.5%)	59 (46.8%)	16 (12.7%)	X^2 ^= 0.12; p = 0.73
	Grade 2 (n = 284)	150 (52.8%)	111 (39.1%)	23 (8.1%)	
	Grade 3 (n = 216)	98 (45.4%)	93 (43.1%)	25 (11.6%)	
**Nodal Involvement**	Absent (n = 434)	214 (49.3%)	179 (41.2%)	41 (9.4%)	X^2 ^= 0.79; p = 0.37
	Present (n = 198)	90 (45.5%)	87 (43.9%)	21 (10.6%)	
**Vascular Invasion**	Absent (n = 473)	224 (47.4%)	201 (42.5%)	48 (10.1%)	X^2 ^= 0.38; p = 0.54
	Present (n = 116)	57 (49.1%)	50 (43.1%)	9 (7.8%)	

**IL1B +3954 C>T**					

**Tumour Grade**	Grade 1 (n = 121)	68 (56.2%)	48 (39.7%)	5 (4.1%)	X^2 ^= 0.053; p = 0.82
	Grade 2 (n = 279)	173 (62%)	90 (32.3%)	16 (5.7%)	
	Grade 3 (n = 215)	131 (60.9%)	70 (32.6%)	14 (6.5%)	
**Nodal Involvement**	Absent (n = 427)	244 (57.1%)	161 (37.7%)	22 (5.2%)	X^2 ^= 0.37; p = 0.54
	Present (n = 194)	120 (61.9%)	61 (31.4%)	13 (6.7%)	
**Vascular Invasion**	Absent (n = 464)	283 (61%)	157 (33.8%)	24 (5.2%)	X^2 ^= 0.43; p = 0.51
	Present (n = 114)	66 (57.9%)	41 (36%)	7 (6.1%)	

**IL1RN +2018 T>C**					

**Tumour Grade**	Grade 1 (n = 125)	55 (44%)	55 (44%)	15 (12%)	X^2 ^= 0.73; p = 0.40
	Grade 2 (n = 280)	150 (53.6%)	107 (38.2%)	23 (8.2%)	
	Grade 3 (n = 215)	108 (50.2%)	86 (40%)	21 (9.8%)	
**Nodal Involvement**	Absent (n = 429)	216 (50.3%)	180 (42%)	33 (7.7%)	X^2 ^= 0.11; p = 0.74
	Present (n = 196)	102 (52%)	72 (36.7%)	22 (11.2%)	
**Vascular Invasion**	Absent (n = 463)	232 (50.1%)	191 (41.3%)	40 (8.6%)	X^2 ^= 1.34; p = 0.25
	Present (n = 118)	67 (56.8%)	42 (35.6%)	9 (7.6%)	

**IL4R +1902 A>G**					

**Tumour Grade**	Grade 1 (n = 137)	87 (63.5%)	44 (32.1%)	6 (4.4%)	X^2 ^= 0.14; p = 0.71
	Grade 2 (n = 308)	195 (63.3%)	97 (31.5%)	16 (5.2%)	
	Grade 3 (n = 228)	146 (64%)	75 (32.9%)	7 (3.1%)	
**Nodal Involvement**	Absent (n = 477)	313 (65.6%)	144 (30.2%)	20 (4.2%)	X^2 ^= 0.11; p = 0.75
	Present (n = 212)	135 (63.7%)	69 (32.5%)	8 (3.8%)	
**Vascular Invasion**	Absent (n = 508)	316 (62.2%)	170 (33.5%)	22 (4.3%)	X^2 ^= 0.002; p = 0.96
	Present (n = 129)	82 (63.6%)	40 (31%)	7 (5.4%)	

**IL6 -174 G>C**					

**Tumour Grade**	Grade 1 (n = 80)	26 (32.5%)	38 (47.5%)	16 (20%)	X^2 ^= 0.04; p = 0.84
	Grade 2 (n = 204)	78 (38.2%)	95 (46.6%)	31 (15.2%)	
	Grade 3 (n = 159)	49 (30.8%)	83 (52.2%)	27 (17%)	
**Nodal Involvement**	Absent (n = 293)	100 (34.1%)	141 (48.1%)	52 (17.7%)	X^2 ^= 0.20; p = 0.66
	Present (n = 143)	52 (36.4%)	67 (46.9%)	24 (16.8%)	
**Vascular Invasion**	Absent (n = 325)	112 (34.5%)	159 (48.9%)	54 (16.6%)	X^2 ^= 0.001; p = 0.98
	Present (n = 85)	29 (34.1%)	42 (49.4%)	14 (16.5%)	

**IL10 -1082 G>A**					

**Tumour Grade**	Grade 1 (n = 80)	23 (28.8%)	37 (46.3%)	20 (25%)	X^2 ^= 0.37; p = 0.54
	Grade 2 (n = 205)	39 (19%)	113 (55.1%)	53 (25.9%)	
	Grade 3 (n = 158)	44 (27.8%)	79 (50%)	35 (22.2%)	
**Nodal Involvement**	Absent (n = 293)	69 (23.5%)	148 (50.5%)	76 (25.9%)	X^2 ^= 0.84; p = 0.36
	Present (n = 143)	38 (26.6%)	73 (51%)	32 (22.4%)	
**Vascular Invasion**	Absent (n = 325)	87 (26.8%)	156 (48%)	82 (25.2%)	X^2 ^= 3.3; p = 0.07
	Present (n = 85)	12 (14.1%)	49 (57.6%)	24 (28.2%)	

**Table 7:** Probable frequencies of the common haplotypes in the interleukin-1 gene cluster in breast cancer and screening control cohorts.

Haplotype*	**Frequency**	**Case, Control Ratios**	**Chi-square**	**P value**
GCCT	0.330	471.5 : 938.5, 321.5 : 674.5	0.352	0.5529
TTCT	0.165	220.7 : 1189.3, 176.8 : 819.2	1.865	0.1720
GCTC	0.133	182.1 : 1227.9, 138.3 : 857.7	0.469	0.4934
GCTT	0.112	169.0 : 1241.0, 99.9 : 896.1	2.239	0.1346
GCCC	0.099	145.8 : 1264.2, 91.3 : 904.7	0.913	0.3392
TCCT	0.032	42.1 : 1367.9, 35.4 : 960.6	0.602	0.4376
TCTT	0.026	32.4 : 1377.6, 30.1 : 965.9	1.202	0.2729
GTCT	0.024	36.1 : 1373.9, 22.6 : 973.4	0.214	0.6440
TTCC	0.022	33.5 : 1376.5, 19.9 : 976.1	0.377	0.5394
TTTT	0.017	21.2 : 1388.8, 19.8 : 976.2	0.817	0.3661
TCCC	0.016	24.6 : 1385.4, 14.1 : 981.9	0.4	0.5271
TCTC	0.013	16.7 : 1393.3, 14.3 : 981.7	0.295	0.5873

* The haplotypes represent the four polymorphisms (IL1A +4845G>T, IL1B +3954C>T, IL1B -511C>T and IL1RN +2018T>C) in that order.

**Table 8:** Studies of the IL1B -511C>T polymorphism in breast cancer.

**Reference**	**Numbers studied**		**Type of controls**	**Susceptibility**	**Severity**	**Ethnicity**
					
	**Cancer**	**Controls**				
Smith KC et al, 2004 [57]	141	261	Bone marrow and solid organ donors – male and female	No association	No association	mixed
Hefler LA et al, 2005 [58]	269	227	Women visiting outpatient dept for various reasons	No association	No association	Caucasian
Our study	703	489	Women with normal mammograms from screening population	No association	No association	British Caucasian

**Table 9:** Studies of the IL1B +3954C>T polymorphism in breast cancer.

**Reference**	**Numbers studied**		**Type of controls**	**Susceptibility**	**Severity**	**Ethnicity**
					
	**Cancer**	**Controls**				
Hefler LA et al, 2005 [58]	269	227	Women visiting outpatient dept for various reasons	No association	No association	Caucasian
Our study	691	420	Women with normal mammograms from screening population	No association	No association	British Caucasian

**Table 10:** Studies of the IL6 -174G>C polymorphism in breast cancer.

**Reference**	**Numbers studied**		**Type of controls**	**Susceptibility**	**Severity**	**Ethnicity**
					
	**Cancer**	**Controls**				
Smith KC et al, 2004 [57]	144	224	Bone marrow and solid organ donors – male and female	No association	No association	mixed
Hefler LA et al, 2005 [58]	269	227	Women visiting outpatient dept for various reasons	OR = 1.5 (1.04–2.3)* for GC vs. GG OR = 2 (1.1–3.6)* for CC vs. GG	No association	Caucasian
Skerrett DL et al, 2005 [59]	88	102	Maternal cord blood samples	No association	No association	mixed
Saha A et al, 2003 [60]	26	95	Unknown	No association	Not studied	Asian Indian
DeMichelle A et al, 2003 [61]	80	0	-	Not studied	C allele associated with 4 year disease free survival (p = 0.02) and overall survival (p = 0.01) in node positive patients	mixed
Iacopetta B et al, 2004 [62]	256	0	-	Not studied	C allele associated with high grade (p = 0.04) and CC genotype associated with poor survival (p = 0.03)	mixed
Our study	497	490	Women with normal mammograms from screening population	No association	No association	British Caucasian

OR: Odds Ratio

**Table 11:** Studies of the IL10 -1082G>A polymorphism in breast cancer.

**Reference**	**Numbers studied**		**Type of controls**	**Susceptibility**	**Severity**	**Ethnicity**
					
	**Cancer**	**Controls**				
Smith KC et al, 2004 [57]	129	223	Bone marrow and solid organ donors – male and female	No association	No association	Mixed
Giordani L et al, 2003 [63]	125	100	Female outpatients without breast cancer	OR = 0.58 (0.32–1.07) for AG vs. AA OR = 0.38 (0.14–0.99) for GG vs. AA	Not studied	unknown
Skerrett DL et al, 2005 [59]	88	102	Maternal cord blood samples	No association	No association	mixed
Wu JM et al, 2005 [64]	87	0	-	Not studied	No association	mixed
Our study	497	498	Women with normal mammograms from screening population	No association	No association	British Caucasian

OR: Odds Ratio
